# Forebrain Organoids to Model the Cell Biology of Basal Radial Glia in Neurodevelopmental Disorders and Brain Evolution

**DOI:** 10.3389/fcell.2022.917166

**Published:** 2022-06-14

**Authors:** Flaminia Kaluthantrige Don, Nereo Kalebic

**Affiliations:** Human Technopole, Milan, Italy

**Keywords:** neural progenitor cells, neural stem cells, neurodevelopmental disease, brain evolution, cerebral organoid

## Abstract

The acquisition of higher intellectual abilities that distinguish humans from their closest relatives correlates greatly with the expansion of the cerebral cortex. This expansion is a consequence of an increase in neuronal cell production driven by the higher proliferative capacity of neural progenitor cells, in particular basal radial glia (bRG). Furthermore, when the proliferation of neural progenitor cells is impaired and the final neuronal output is altered, severe neurodevelopmental disorders can arise. To effectively study the cell biology of human bRG, genetically accessible human experimental models are needed. With the pioneering success to isolate and culture pluripotent stem cells *in vitro*, we can now routinely investigate the developing human cerebral cortex in a dish using three-dimensional multicellular structures called organoids. Here, we will review the molecular and cell biological features of bRG that have recently been elucidated using brain organoids. We will further focus on the application of this simple model system to study in a mechanistically actionable way the molecular and cellular events in bRG that can lead to the onset of various neurodevelopmental diseases.

## Study of Human Development in a Dish

The temporal series of events that leads to the acquisition of specific structural and functional features of different organs in the human body is a fascinating, yet not fully understood phenomenon known as organogenesis. Heart, brain, skin and liver are all very distinct and specific organs with their own distinct functions, yet they developmentally originate from a single cell. Unveiling the cascade of steps leading from such a simple disordered system to an ordered complexity is not only essential from a developmental biology perspective but also for establishing therapeutic approaches in the context of regenerative medicine.

Whereas the classical *in vivo* model systems, such as Drosophila, zebrafish and mouse provided fundamental insight into the basic animal, vertebrate and mammalian development respectively, certain aspects of the complexity observed in humans can only be studied in the human model system. Hence, 2D *in vitro* and *ex vivo* systems, such as organotypic cultures, have been valuable to reveal human-specific features of organ development and pathology (Shamir and Ewald, 2014). However, they lack either the spatial complexity of the tissue or the ability to study the development for prolonged time periods. This raised the need to establish a human model system that would mimic human organogenesis with a sufficient level of spatio-temporal complexity.

The first step towards this goal was provided by the pioneering work of somatic cells reprogramming into pluripotent stem cells (PSCs) (Takahashi and Yamanaka, 2006). The subsequent ability to grow PSCs enabled the exploitation of this technology to generate human stem cell derived cultures (Takahashi et al., 2007). Additionally, cultivation of PSCs in a 3D configuration enabled the cell-cell and cell-extracellular matrix (ECM) communication which would otherwise be absent in a 2D culture (Blau and Miki, 2019). In 2008 a remarkable work conducted by Eiraku and others established for the first time a 3D polarised cortical tissue from embryonic stem cells (ESCs) (Eiraku et al., 2008). This paved the road towards the use of organoids, as multicellular structures that exhibit the capacity to self-organise into a complex system, to study organogenesis in a dish. The term “organoid” was consolidated by Sato and others who established for the first time intestinal organoids from single adult stem cells (Sato et al., 2009). Together, these fundamental studies led to the widespread application of organoid technology in developmental biology (Kaluthantrige Don and Huch, 2021).

Organoids contain the genetic background along with the cell-cell and cell-ECM interactions similar to those *in vivo*, however, they are grown in a simpler and fairly controllable environment (Shamir and Ewald, 2014). On the one hand this is a limiting factor in recapitulating the physiological features of organogenesis. On the other hand, this is an opportunity to dissect in depth the biology of cells of interest within an environment that can be readily controlled. For instance, hepatocyte organoids, that recapitulate the spectacular ability of the liver to regenerate upon a partial resection, can be used as a magnifying glass to study the cell types underlying this regeneration (Hu et al., 2018; Peng et al., 2018). In the context of human pathologies, organoids hold potential to treat various diseases, such as acute kidney injury or diabetes (Lancaster and Huch, 2019). Recently, kidney organoids transplanted under the renal capsule of mice acquired *de novo* vascularisation and tubular maturation (van den Berg et al., 2018), allowing future applications for the treatment of renal failure. Furthermore, the use of pancreatic islet organoids as a source of β-cells *in vitro* may potentially be an alternative cell therapy for diabetes (Wang et al., 2020). Comparison between human fetal retina and retinal organoids showed considerable similarities, thus anticipating a potential role of retinal organoids as cell source for transplantation in cell therapy (Sridhar et al., 2020).

A striking example of a model system that successfully simplifies a highly complex organ, but however mimics the key aspects of the development, is a brain organoid (see section 1.3 for discussion on brain organoids) (Heide et al., 2018; Benito-Kwiecinski and Lancaster, 2020; Lopez-Tobon et al., 2020; Marton and Pașca, 2020; Velasco et al., 2020; Sidhaye and Knoblich, 2021). Untangling the functional dynamics of distinct brain cells using animal models is a very tedious process due to the remarkable complexity stemming from the interaction of the brain with the other organs and the environment. One fundamental question is to understand how this complexity arises during development.

## Basal Radial Glia - A Key Cell Type for Human Neocortex Development

The cerebral cortex, and its evolutionary most recent part, the neocortex, arise from the forebrain region of the neural tube. It is arguably considered that the higher cognitive abilities of humans compared to other mammals are reflected by the size and the cytoarchitectural organisation of the human neocortex (Molnár et al., 2019; Rakic, 1988, 1995). Development of the neocortex initiates with the proliferation of neuroepithelial cells lining the neural tube. Transition from a proliferative state into a neurogenic state gives rise to apical radial glia (aRG), the chief parental progenitor cells that will initiate the neurogenesis, that is the series of events that lead to the production of neurons (Götz and Huttner, 2005; Taverna et al., 2014). The identity of the progenitors is defined based on the location of their mitosis (Taverna et al., 2014), which highlights the importance of the microenvironment for the cell fate specification. Indeed, proliferation of aRGs occurs on the ventricular (apical) surface and these cells form the apical-most neocortical histological layer, known as the ventricular zone (VZ) (Götz and Huttner, 2005). Moving along the apicobasal axis, asymmetrical divisions of aRGs give rise to basal progenitors that populate the second germinal layer, the subventricular zone (SVZ). In species with an expanded neocortex, the SVZ is divided into two distinct zones: the inner and the outer SVZ (ISVZ and OSVZ, respectively) (Smart, 2002; Dehay et al., 2015).

The neocortical expansion in mammals has been widely associated with a subpopulation of basal progenitors called basal or outer radial glia (bRG or oRG) (Fietz et al., 2010; Hansen et al., 2010; Reillo et al., 2011). The abundance of bRG and their proliferative capacity are strikingly increased in species with an expanded neocortex, such as human, macaque or ferret (Fietz et al., 2010; Hansen et al., 2010; Reillo et al., 2011; Betizeau et al., 2013; Kalebic et al., 2019), compared to species with a small brain, such as mouse (Wang et al., 2011; Wong et al., 2015). This results in an increased production of neurons, which in turn is associated with the expansion and folding of the neocortex. Hence, bRG are considered to be a key cell type underlying human neocortex development (Penisson et al., 2019; Kawaguchi, 2020; Pinson and Huttner, 2021; Del-Valle-Anton and Borrell, 2022). An additional layer of cellular complexity within the bRGs lies in their morphological heterogeneity (Kalebic and Huttner, 2020). We have identified that an increasing number of basal processes within the human bRGs coincides with an increase in their proliferative capacity (Kalebic et al., 2019). Interestingly, such bRGs complexity and proliferative capacity are absent in the mouse cortex, further corroborating the role of bRGs as chief cells underlying mammalian neocortical expansion.

As many studies have started to focus on this fascinating population of cells, multiple outstanding questions remain to be addressed. For example, what is the molecular and cell biological heterogeneity of bRG across different species and what is their contribution to the onset of human intellectual disabilities. The use of a reductionist system containing abundant bRGs such as the organoids may elucidate the mechanisms that lead towards the complexity of the neocortex organogenesis and pathogenesis. This review will focus on the diverse modalities to generate brain organoids and how we can exploit this technology in the context of neocortex development and pathologies, specifically focusing on bRGs.

## Forebrain Organoids

During embryogenesis, the interplay of diverse signalling pathways leads to the differentiation into neuronal fate. Initial inhibition of the the bone morphogenic proteins (BMP) signalling is needed for the differentiation into the neuroectoderm which then invaginates to give rise to the neural tube (Muñoz-Sanjuán and Brivanlou, 2002; Sadler, 2005). Patterning of the neural tube into different regional identities is achieved through the regulated activity of WNT, fibroblast growth factor and retinoic acid pathways (Muñoz-Sanjuán and Brivanlou, 2002; Molotkova et al., 2005). To model such embryonic development *in vitro* and building on the earlier pioneering work, two studies reported the generation of brain organoids in 2013 (Kadoshima et al., 2013; Lancaster et al., 2013). Two distinct approaches have been applied for generating brain organoids: 1) the unguided method, which directs the generation of organoids with multiple regional identities; and 2) the guided method, which promotes the acquisition of specific regional identity through a step-wise time-dependent exogenous signalling (Table 1) (Lancaster and Knoblich, 2014; Di Lullo and Kriegstein, 2017). Each approach starts with the generation of 3D aggregates named embryoid bodies (EBs), which have the potential to differentiate into all three germ layers (Zhang et al., 2001). The first cerebral organoids were generated from EBs following the intrinsic program of neuroepithelial cells to differentiate into neural progenitor cells (Lancaster et al., 2013). Such unguided protocol results in a stochastic development of various and multiple regional identities (Ormel et al., 2018). Alternatively, the differentiation can be directed towards the acquisition of a specific regional identity, such as the dorsal forebrain. The latter can be achieved through the manipulation of the transforming growth factor-beta (TGF-β) signalling pathway and BMPs (Chambers et al., 2009; Kadoshima et al., 2013; Paşca et al., 2015; Qian et al., 2018, 2020; Sloan et al., 2018). Of note, the time dependent addition of small molecules in presence (Kadoshima et al., 2013; Lancaster et al., 2013; Qian et al., 2018, 2020; Sloan et al., 2018) or absence (Mariani et al., 2015) of Matrigel, a commercially available basement membrane matrix from mouse sarcoma (Li et al., 1987), shows a remarkable difference in the developmental timelines, prompting a question on how it might affect the progenitor biology (Table 1).

**TABLE 1:** Brain organoid protocols.

Organoid protocol	Method (Guided or unguided)	Cell line	EB generation	Matrigel	Bioreactor	Orbital shaker	Slicing
Kadoshima et al. (2013)	guided	ESCs	From single cells 96 WP		✗	✗	✗
				✓			
				From day 35 (Matrigel 1% vol/vol)			
				From day 70 (Matrigel 2% vol/vol)			

Lancaster et al. (2013)	unguided	ESCs: H9	From single cells	✓	✓	✗	✗
		H1	96 WP	From day 11 in Matrigel droplets			
Giandomenico et al. (2021)	guided	ESCs	From single cells	✓	✓	✓	✓
		H9	96 WP				Only once between
		H1	with microfilaments				day 45 and 60
Pasca et al. (2015)	guided	iPSC	From single cells	✗	✗	✓	✗
			96 WP				
Qian et al. (2020)	guided	iPSC	From whole colonies	✓	✓	✓	✓
			Use of collegenase to	During the forebrain patterning	Optional during differentiation	During differentiation and maturation	Day 45 - Day 150
			lift the colonies 6WP	day 7–14	Day 14–72	Day 45–150	Once a month

Abbreviations: EB, embryoid body; ESCs, embryonic stem cells; iPSCs, induced pluripotent stem cells.

**FIGURE 1:**
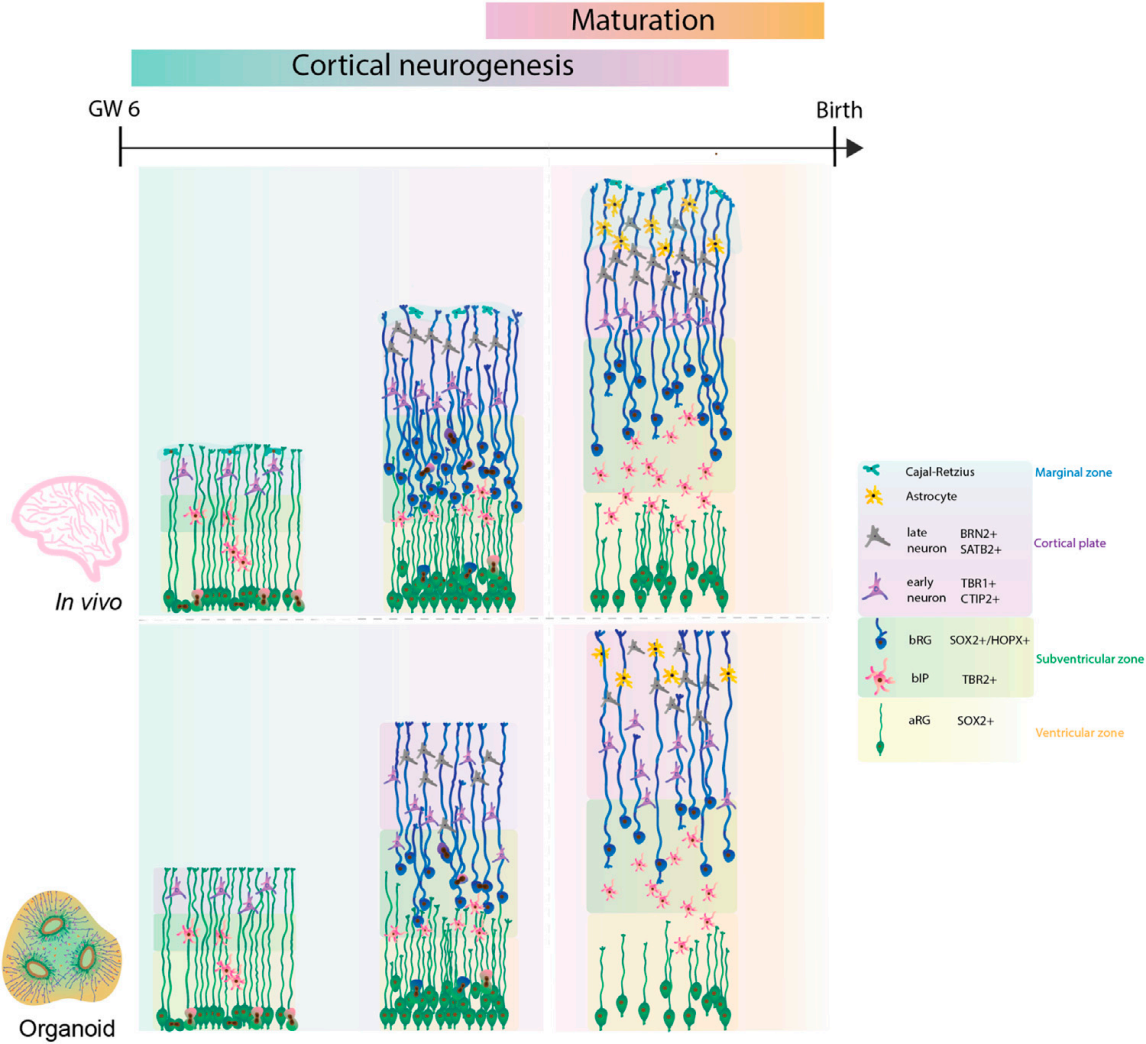
Comparison of human cortical development between human fetal neocortex and human forebrain organoids. Cortical neurogenesis in human fetal development (upper panel) starts with proliferation of neural progenitor cells. In this process, bRG are highly abundant and give a key contribution to the final neuronal output. Consequently, the neocortex expands into specific cytoarchitectural layers with formation of cortical folding on the basal side. Following neurogenesis, functional maturation of neurons and glia takes place. Human forebrain organoids (lower panel) recapitulate the cell diversity and developmental lineages, however further improvements are required to achieve bRG abundance, improved cytoarchitectural organisation, neuronal and glial maturation, and cortical folding similar to the ones observed during the human fetal neocortex.

Although the timeline in the induction of neurogenesis is different, all protocols partially recapitulate the series of events known to occur in the developing human neocortex (Figure 1). Indeed, organoids readily contain aRGs marked by the expression of the transcription factors SOX2 and PAX6 (Kadoshima et al., 2013; Lancaster et al., 2013; Qian et al., 2018, 2020). Both subtypes of basal progenitors, bRG and the intermediate progenitors can be observed in organoids. bRG are marked by the presence of HOPX, SOX2 and PAX6, along with the absence of TBR2 (Pollen et al., 2015; Qian et al., 2016, 2020). Moreover, organoids also display the presence of early born (CTIP2+ or TBR1+) and late born neurons (SATB2+ and/or BRN2+) (Figure 1) (Paşca et al., 2015; Lancaster et al., 2017; Sloan et al., 2018; Qian et al., 2020; Giandomenico et al., 2021). Transcriptomic analysis identified that mature brain organoids between days 250 and 300 correspond to postnatal stages of human brain development (Gordon et al., 2021). However, the timing of expression of different cell types and formation of specific cortical layers differs from protocol to protocol. For example, the organoids produced by the Lancaster protocol show the presence of neurons already at day 30 (Lancaster et al., 2013). This contrasts the organoids generated by the Kadoshima protocol, which start neurogenesis after 70 days (Kadoshima et al., 2013). Thus, the caveat of timing across protocols needs to be considered when comparing the fetal human development.

Most of the initial organoid protocols showed similar limitations as they poorly recapitulated the tissue architecture, notably cortical layering, the bRG abundance, the presence of all developmental lineages and neuronal maturation. To address these limitations and to improve the nutrient and oxygen exchange within the organoid, several improvements of the initial protocols were reported. For example, adding microfilaments and culturing organoids at air-liquid interfaces advanced the original Lancaster protocol (Lancaster et al., 2017; Giandomenico et al., 2021). Recently, the use of external magnetic nanoparticles or inclusion of signalling gradients showed enhanced local patterning of brain organoids (Cederquist et al., 2019; Fattah et al., 2022). It is tempting to speculate that such methods could direct an improved cytoarchitectural organisation of forebrain organoids (Cederquist et al., 2019; Fattah et al., 2022). Further, repeated slicing of Qian organoids facilitated an expansion of cortical layers and an increased expression of HOPX + bRG cells at day 80, reminiscent of the OSVZ (Qian et al., 2020).

Another strategy to improve brain organoid maturation resulted in the fusion of phenotypically independent dorsal and ventral organoids, termed assembloid (Bagley et al., 2017; Sloan et al., 2018). The latter is particularly interesting because the ventral part of the forebrain is the principal origin of human interneurons, that subsequently migrate into the dorsal regions to integrate into the cortical circuits (Bandler et al., 2017; Hu et al., 2017; Lim et al., 2018). Interneurons are generated by the radial glia of the ventral forebrain, which appear to be more similar to the dorsal aRG than bRG (Velmeshev et al., 2021). Taken together, dorsal-ventral assembloids provide a model system to study generation, migration and integration of interneurons, which enables a more complete modelling of the human cortical development (Marton and Pașca, 2020).

Finally, several strategies have been adopted to improve vascularization. One approach consists in the co-culture of brain organoids with vascular cells such as human umbilical vascular endothelial cells (HUVECs). This resulted in a reduced hypoxic core and improved neuronal maturation (Shi et al., 2020). Additional strategy transplanted organoids into vascularised tissue of immunodeficient mice and showed functional blood circulation and improved organoid viability (Mansour et al., 2018). Implementation of vascularisation in forebrain organoids could enhance the viability and potentially promote neuronal maturation.

## What Have Organoids Told us About bRG?

As mentioned above, bRG are considered to be the key cell type underlying human neocortical development. Human bRG are highly proliferative, likely generate most of the human neurons and serve as the scaffold for the neuronal migration to the cortical plate (Fietz et al., 2010; Hansen et al., 2010; Reillo et al., 2011; LaMonica et al., 2013). bRGs biology has been poorly assessed since the abundance and behaviour of this cell type is strikingly low in the key animal model, the mouse (Wang et al., 2011; Wong et al., 2015). Although the abundance of bRGs in cerebral organoids is still not comparable to the numbers present in fetal human tissue, organoids hold great promise to be a suitable *in vitro* system to study human bRGs.

### Molecular Characterization of bRG

To understand the extent at which the organoid system recapitulates the bRG identity observed in human fetal tissue, initial studies examined the transcriptomic profiles of both model systems. This revealed similar lineage relationships between aRG and bRG in both systems (Camp et al., 2015; Pollen et al., 2015). Subsequent studies readily confirmed the existence of a cell population with a transcriptomic signature of bRG (Bershteyn et al., 2017; Giandomenico et al., 2019; Pollen et al., 2019; Velasco et al., 2019; Cheroni et al., 2022). A recent work aimed to understand the reproducibility of organoids, identified consistent generation of diverse cell types, including bRG, in multiple forebrain organoids (Velasco et al., 2019). Subsequent work combined the latter dataset with spatial transcriptomics and identified a spatial patterning of different cell types within the organoid, with bRG being superficially positioned with respect to aRG (Uzquiano et al., 2022).

One striking characteristic of brain organoids compared to other organoids, such as the liver, is their outstanding increase in size during maturation. However, this can result in poor oxygenation and nutrient exchange within the organoid core causing a systematic cellular stress (Bhaduri et al., 2020). A recent analysis, however, suggested that the cellular stress is a feature of a subpopulation of cells, which can be removed in subsequent computational analyses (Vértesy et al., 2022).

Additional transcriptomic studies extended the role of brain organoids not only as a promising tool to tackle human cortical development but also showed the valuable use of organoids in modelling human brain evolution (Heide et al., 2018; Muchnik et al., 2019). For example, *CTCL*, a fusion transcript and a Wnt signalling modulator, which is expressed in human but not mouse developing brain, has recently been shown to be implicated in the proliferative capacity of bRG in human organoids (Ou et al., 2021). Comparison between human and non-human primate brain organoids pointed at the increased activation of another key signalling pathway, the PI3K-AKT-mTOR, in human bRG (Pollen et al., 2019; Andrews et al., 2020). Moreover, mTOR signalling in human organoids was shown to regulate bRG morphology and behaviour (Pollen et al., 2019; Andrews et al., 2020). Building on earlier findings that identified the role of Notch signalling in promoting human bRGs proliferation (Hansen et al., 2010), recent work conducted in brain organoids, identified the role of a human-specific gene *NOTCH2NL* to enhance the activity of Notch signalling and to delay the neural differentiation of bRG (Fiddes et al., 2018). The second human-specific gene implicated in neocortical expansion and known to operate in bRG, ARHGAP11B (Florio et al., 2015; Kalebic et al., 2018; Heide et al., 2020), has been introduced to chimpanzee organoids where it promoted bRG proliferation (Fischer et al., 2020). Kanton and others performed a comprehensive cell-type specific analysis of gene expression in human, chimpanzee and macaque organoids and further revealed the molecular mechanisms underlying the differences in gene expression across these species (Kanton et al., 2019; Muchnik et al., 2019). Their ATAC-seq analysis showed divergence in chromatin accessibility between human and chimpanzee organoids, which could be associated with the human-specific gene expression (Kanton et al., 2019; Muchnik et al., 2019). Additionally, organoids also offer the possibility to compare the differences in brain development between modern humans and ancestral species such as Neandertals. For instance, Muotri and others modelled Neandertal brains in organoids by introducing an archaic variant gene called Neuro-oncological ventral antigen 1 (NOVA1) (Trujillo et al., 2021). These organoids exhibited changes in organoid morphology and neuronal activity (Trujillo et al., 2021). Recently, Mora-Bermudez and others introduced specific ancestral variants involved in mitotic spindle and kinetochore function in organoids and showed shorter metaphase of apical progenitors compared to the longer metaphase of non-mutated modern human organoids (Mora-Bermúdez et al., 2022). It is interesting to speculate that such evolutionary differences between modern humans and ancestral human species might also be linked to bRG development and function.

### Cell Biology of bRG

**FIGURE 2:**
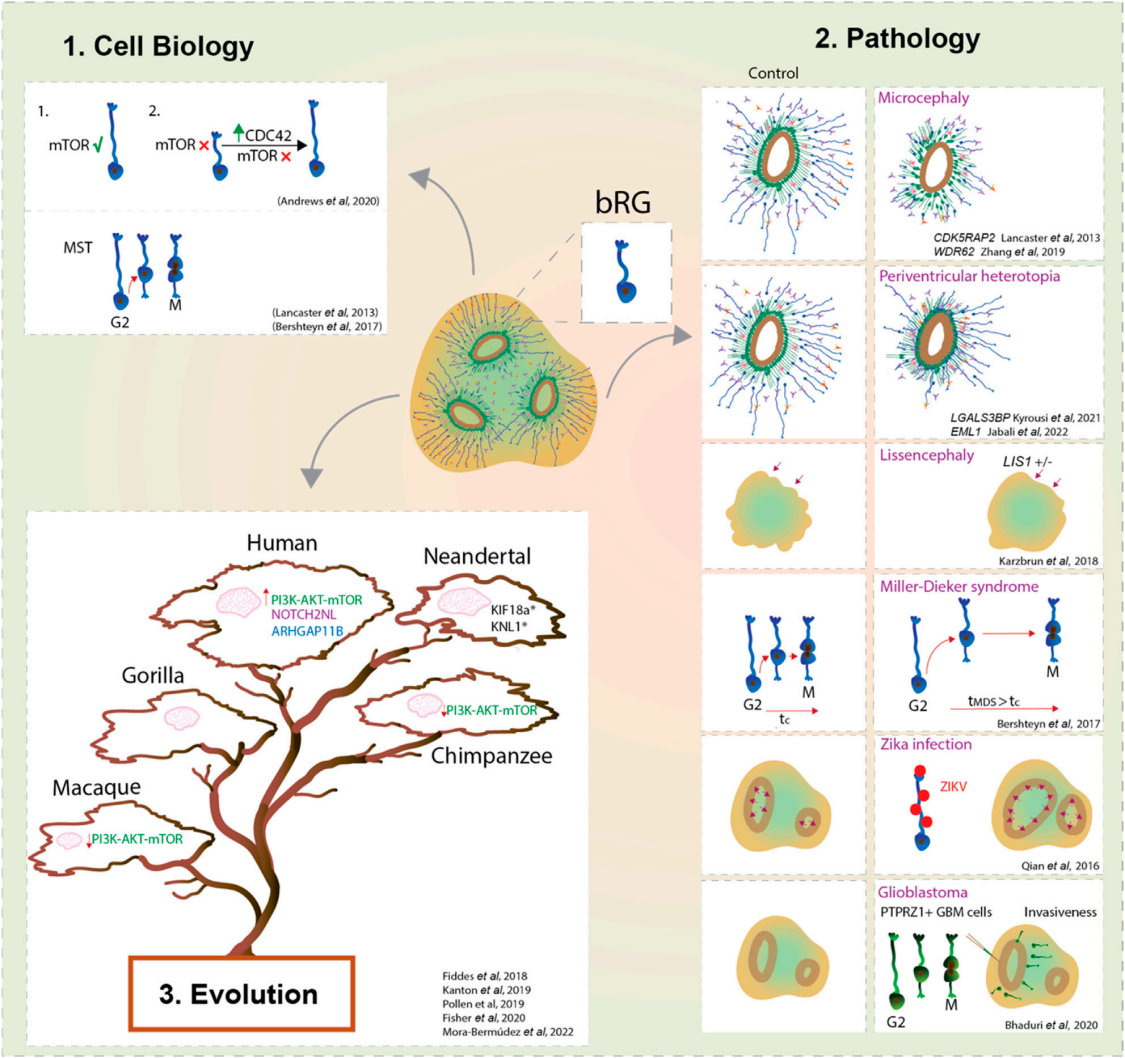
bRG in development, evolution and pathology using forebrain organoids. Forebrain organoids enable us to study the cell biological features and cell behaviours that characterise bRG (1); their role in the onset of malformations of cortical development, brain cancers and viral diseases (2); and their contribution to the neocortex expansion of modern humans compared to ancestral human species and non-human primates (3). Overall, organoids provide a new window into bRG and their link with the expansion of the neocortex.

The transcriptomic studies described above have shown that organoids successfully recapitulate the diversity of cell types and their lineage relationships. Combined with the existence of the organoid polarity, it allows us to use this model system to also study the cell biological features of bRG (Figure 2). Previous identification on the role of mTOR signalling pathway (Nowakowski et al., 2017) in human bRG led to its deeper analysis using organoids (Pollen et al., 2019). Upon the pharmacological inhibition of mTOR signalling in organoids, bRG exhibited a shorter basal process (Andrews et al., 2020). Interestingly, bRG morphology could be rescued by the activation of the Rho-GTPases CDC42/RAC1 in cortical tissue (Figure 2) (Andrews et al., 2020). CDC42 in particular has a very important role in radial glia morphology, as it has been found to affect polarisation, proliferation and migration of aRG (Cappello et al., 2006; Yokota et al., 2010). Another determinant of cell polarity involved in maintenance of the radial scaffold is GSK3 (Yokota et al., 2010). Recently, a pharmacological inhibition of GSK3 in organoids led to a reduction in the abundance of bRG and production of neurons, further emphasising the role of bRG polarity for normal neurogenesis (López-Tobón et al., 2019). Although bRG lack classical apicobasal polarity and the contact with the ventricular surface, they do possess a series of features that we have previously termed pseudo-apicobasal polarity (Kalebic and Namba, 2021). A key manifestation of such polarity is their morphology. Previous findings identified different bRG morphotypes in mouse, ferret, macaque and human developing cortex (Betizeau et al., 2013; Reillo et al., 2017; Kalebic et al., 2019), suggesting that an increased number of basal processes coincides with the proliferative capacity of bRG (Kalebic and Huttner, 2020). It would be interesting to identify these diverse bRG morphotypes in organoids and understand how morphology might be an important component for neurogenesis progression.

Another key advantage of the organoid system is that it enables the studies of bRG structural and temporal dynamics without the complexity inherent to the *in vivo* and *ex vivo* systems. Prior to mitosis, bRG exhibit a distinctive saltatory migrational behaviour named mitotic somal translocation (MST) (Hansen et al., 2010; LaMonica et al., 2013; Ostrem et al., 2014; Ostrem et al., 2017). Remarkably, organoid studies based on GFP-electroporated radial glia identified this unique feature in cells localised away from the VZ (Lancaster et al., 2013; Otani et al., 2016) The importance of MST for human cortical development is obvious when observing a form of human lissencephaly called Miller-Dieker syndrome (MSD). MSD brain organoids showed prolonged mitosis and longer MST distances (Figure 2) (Bershteyn et al., 2017), suggesting that defects in bRG mode of division could lead to premature neurogenesis in human lissencephaly.

### bRG in Pathology

Organoids, especially when derived from patients’ cells, are a par excellence platform to dissect the pathogenesis of neurodevelopmental diseases (Figure 2). One of the first examples comes from the work conducted by Lancaster and others who generated patient-derived microcephalic cerebral organoids carrying a mutation in the centrosomal protein CDK5RAP2 (Lancaster et al., 2013). They showed an increase of asymmetric cell divisions in neural progenitors which led to their premature differentiation (Lancaster et al., 2013). The second principal way to model neurodevelopmental disorders in organoids is to introduce disease-causing mutations *via* genome editing in PSCs. For example, deletion of *WDR62*, another key gene causing microcephaly, resulted in a reduction of bRG proliferation, which in turn led to reduced organoid size (Zhang et al., 2019).

In addition to microcephaly, organoids have been useful to model periventricular heterotopia (Figure 2). A recent study used both patient-derived and genome-edited PSCs to study EML1 deficiency in cortical organoids (Jabali et al., 2022). The analyses revealed defects in the primary cilium structure and mitotic spindle orientation of aRG, which led to an increase in aRG delamination and subsequent formation of ectopic neural progenitors and heterotopic neurons (Jabali et al., 2022). Interestingly, deeper characterization identified that the majority of these ectopic progenitor cells are bRG with an unusual morphology (Jabali et al., 2022), linking bRG morphology with neurogenesis. Phenotypes of periventricular heterotopia were successfully recapitulated in human brain organoids also by manipulation of the expression levels of *ECE2* and *PLEKHG6* (O’Neill et al., 2018; Buchsbaum et al., 2020). Another key gene that has been recently described to be enriched in human bRG (Pollen et al., 2015), while being linked to periventricular nodular heterotopia, is *LGALS3BP*. Studies using organoids showed that *LGALS3BP* expression is essential for proper positioning of bRG, whereas altered *LGALS3BP* expression resulted in neuronal heterotopia and defects in local gyrification, emphasising once again a potential role of bRG in disease (Kyrousi et al., 2021).

Further studies identified a role of bRG in the pathogenesis of Pretzel syndrome (polyhydramnios, megalencephaly, symptomatic epilepsy; PMSE) derived from mutations in the STRADA gene, part of the mTOR pathway. PMSE organoids showed an increase of HOPX + bRG which could be linked with the megalencephaly observed in PMSE individuals (Dang et al., 2021). This also further strengthens the role of the mTOR pathway in the regulation of bRG (Nowakowski et al., 2017; Andrews et al., 2020). Mutation of *CHD8* (chromodomain helicase DNA-binding 8) in cerebral organoids resulted in an increased proliferation of a population of radial glial cells which translated into altered neurodevelopmental trajectories (Villa et al., 2022).

Several studies modelled cortical folding using organoids (Figure 2). Activation of the PI3K-AKT signalling is known to be involved in increased proliferation of BPs (Kalebic et al., 2019) and its dysfunction is associated with brain overgrowth disorders (Hevner, 2015). Genetic ablation of *PTEN,* a regulator of PI3K, in human organoids showed an increase of HOPX + bRG with subsequent formation of cortical folding (Li et al., 2017). Interestingly, both *PTEN* mutant mice and human organoids showed an increase of brain or organoid volume, but only human organoids showed folding (Li et al., 2017). This suggests the importance of specific molecular and/or cellular features in humans, but not in mice, to direct cortical folding. Nevertheless, control human brain organoids lack the ability to achieve cortical folding, suggesting that they exhibit insufficient neuronal maturation and/or lack the mechanical signals from the microenvironment (Borrell, 2018; Kroenke and Bayly, 2018). Gyrification is important for the development of the neocortex as it maximises the surface to pack neurons relative to the brain size. Karzbrun et al. reported an induction of folding in organoids by physically constraining brain organoids using a chip (Karzbrun et al., 2018). Together with the mechanical forces from the cytoskeleton contraction and cell migration this induced wrinkles in organoids that are reminiscent of cortical folding (Karzbrun et al., 2018). In contrast, lissencephalic organoids (*LIS-1* mutant) showed changes in the cytoskeleton and ECM that resulted in a reduced organoid wrinkling (Karzbrun et al., 2018). It would be interesting to apply the chip device to organoids whose age corresponds to the onset of bRG neurogenesis and examine a link between bRG and the mechanisms of cortical folding. Interestingly, in lissencephalic organoids modelling Miller-Dieker syndrome, bRG showed mitotic defects, suggesting a role of bRG in pathogenesis of lissencephaly (Bershteyn et al., 2017).

Apart from neurodevelopmental disorders, the use of brain organoids was beneficial to elucidate a role of bRG-like cells in malignant brain tumours such as glioblastoma (Figure 2). Live imaging on primary resected tumours displayed a population of bRG-like cells undergoing MST (Bhaduri et al., 2020). Upon transplantation into cortical organoids, these cells exhibited typical invasiveness and expansion of tumour-like cells (Bhaduri et al., 2020). This highlights an important role of bRG biology not only during brain development but also in the context of cancer progression.

Finally, brain organoids have a potential to mimic viral infectious diseases (Figure 2) (Harschnitz and Studer, 2021). An outstanding example was given in response to the outbreak of Zika virus (ZIKAV), in which ZIKAV induced microcephalic organoids were generated (Qian et al., 2016; Krenn et al., 2021). These organoids exhibited increased apoptosis, reduced proliferation with subsequent reduction of organoid size. Interestingly, the authors showed that bRG were readily infected by the Zika virus (Qian et al., 2016), hence indicating the advantage of using organoids to understand the contribution of different cell types, such as bRG, in the disease aetiology (Figure 2).

Brain organoids have hence provided invaluable insight into the role of bRG for human neurodevelopmental pathologies. Since rodent models poorly recapitulate features of human bRG, ferrets and primates are typical species of choice for *in vivo* exploration of the bRG role in neurodevelopmental disorders (Feng et al., 2020; Gilardi and Kalebic, 2021). Although they recapitulate well the key aspects of bRG biology, these models require substantial time and resources in addition to important ethical considerations for disease modelling. Hence the application of organoids, and particularly patient-derived organoids, has been instrumental for the advancement of knowledge regarding neurodevelopmental diseases and role of bRG in this context.

## Conclusion

The ability to recapitulate organogenesis outside the embryo makes the organoid system a fascinating and useful technology. Although brain organoids differ from the brain *in vivo*, their ability to reproduce the diverse cell types and lineage trajectories comparable to human fetal cortex, makes the organoids a promising tool to address fundamental questions in neocortical development and pathologies. This is particularly relevant for bRG, a key progenitor cell type underlying human brain development. Future research will likely focus on further cell biological characterization of bRG in organoids and will better dissect the steps along the developmental trajectories examining the contribution of bRG for neocortical development. Given that organoids are becoming a key model system to study differences in brain development between modern humans and ancestral species, it is likely that further efforts in this direction will elucidate the contribution of specific genetic changes between these species for the biology of basal progenitors. Finally, disease modelling has been one of the principal directions of organoid-based research. Future efforts in this domain are expected to further develop in the direction of an ever-more personalised medicine combining patient-derived organoids with genetic and pharmacological screens. An elegant example of a genetic screen has been performed by Esk and others who tested 173 microcephaly-related genes in human brain organoids using CRISPR/Cas9-mediated genome editing (Esk et al., 2020). Future approaches can be used to study candidate genes of other neurodevelopmental pathologies, genes that have more subtle differences in expression level between control and pathological development and, finally, genes whose phenotype is likely to be pertinent to the later stages of organoid development, when bRG become more dominant. Hence, although brain organoids still do not recapitulate all the features of human cortical development, further advancement of the technology and/or combination with xenografting into animal models, are likely to pave the way for an ever-increasing use of this model system to study neurodevelopmental pathologies and human brain evolution.

## Abbreviations

aRG, apical radial glia; bIP, basal intermediate progenitor; bRG, basal radial glia; GW, gestational week.
